# The neuroprotective effect of ascorbic acid against imidacloprid-induced neurotoxicity and the role of HO-1 in mice

**DOI:** 10.3389/fneur.2023.1130575

**Published:** 2023-04-20

**Authors:** Rajat Mudgal, Satyam Sharma, Sanjiv Singh, V. Ravichandiran

**Affiliations:** Department of Pharmacology and Toxicology, National Institute of Pharmaceutical Education and Research, Hajipur, Bihar, India

**Keywords:** HO-1/Nrf2 pathway, neurotoxicity, ascorbic acid, imidacloprid, pesticide

## Abstract

Imidacloprid (IMI) is not only a neurotoxic agricultural pesticide but also a possible food contaminant. The aims of this study were to (1) explore the relationship between recurrent IMI administration and neuronal toxicity in mice and (2) evaluate the potential neuroprotective effect of ascorbic acid (AA), a substance with significant free radical scavenger and having property to block the inflammatory pathways. Mice were categorized as naïve controls (administered vehicles for 28 days); the IMI-treatment animal group (administered po 45-mg/kg body weight of IMI per day for 28 days); and the IMI + AA treatment animal group (administered the same IMI dose + 200 mg/kg of AA orally for 28 days). On day 28, memory losses were assessed using the Y-maze and novel target identification behavioral tests. Mice were sacrificed 24 h after the final IMI treatments, as well as hippocampus tissues, were utilized to determine histological assessments, oxidative stress biomarkers, and Heme oxygenase-1 (HO-1) and nuclear factor erythroid 2-related factor 2 (Nrf2) gene expression levels. The findings demonstrated that IMI-treated mice had substantial impairment of spatial and non-spatial memory functions, as well as reduced antioxidant enzyme and acetylcholinesterase activity. The AA neuroprotective action was achieved through the suppression of the HO-1 expression as well as the stimulation of Nrf2 expression in hippocampal tissues. In summary, recurrent IMI exposure causes oxidative stress and neurotoxicity in mice, and the administration of AA significantly reduces the IMI toxicity possibly by the activation of the HO-1/Nrf2 pathway.

## Introduction

Imidacloprid (IMI) is a neonicotinoid insecticidal compound that is mostly consumed by farmers in farming around the world (1). IMI residues were found in fruits and vegetables, cereals, and water supplies on a regular basis (2). Based on the dose and exposure timing, IMI causes different types of toxicity, such as hepatotoxicity, nephron toxicity, and neuronal toxicity (3). Repeated exposure to IMI, in particular, has been suggested to cause a varying ratio of neuronal toxicity, including learning and memory impairment, neurodegenerative disorder development, axonal flow impairments, and diminished (BBB) blood-brain barrier integrity (4, 5). The suppression of acetylcholinesterase activity in the hippocampus contributes to IMI toxicity (6). The activation of the oxidative stress pathways is one of the molecular routes by which IMI impacts behavior and cognition (7), indicating antioxidants as possible treatments for IMI-induced oxidative stress and its deleterious effects.

Ascorbic acid (AA) ((2R)-2-[(1S)-1,2-dihydroxyethyl] ascorbic acid-3,4-dihydroxy-2H-furan-5-one) is a water-soluble antioxidant that is present in a variety of plants, such as broccoli, citrus fruits, blackcurrants, and strawberries (8). Several studies have shown that AA has antioxidant and anti-inflammatory properties (9). Numerous disorders, including diabetes, allergies, cardiovascular, neurodegenerative, and infectious diseases have been reported to benefit from AA (10–13). AA not only protects against the onset of several neurodegenerative disorders, including Parkinson's disease and Alzheimer's disease, but is also useful in neuronal damage caused by ischemic and neuronal inflammation conditions (14, 15). Apart from its ability to inhibit oxidative stress-induced apoptosis, AA's neuroprotective properties are mostly due to its antioxidant activity (16). Ascorbic acid is neuroprotective against sciatic nerve injury in rats (17), as an antioxidant ascorbic acid attenuates oxidative cell death (18), causes apoptosis (19), and ameliorates intracellular reactive oxygen species and inflammatory biomarkers (20). Ascorbic acid has proven to have neuroprotection against ischemia and excitotoxicity *via* various pathways (21–23). Vitamin C protected the postnatal rat brain from ethanol-induced neurodegeneration; in addition, neuroglial cells from ethanol caused toxic effects (24). Ascorbic acid attenuated the lead-induced apoptosis in the hippocampus (25).

Heme oxygenase-1 (H0-1) is a kinase enzyme that is potentiated by prolonged oxidative stress in hippocampus cells (26). It is highly associated with the neuronal pathology of neurological impairments since HO-1 hyperactivation is directly linked with elevated tau phosphorylation, neurofibrillary tangles, and increased formation of Aβ (27, 28). One of the transcription factors that protect cells from oxidative damage is Nrf2 which protects cells from oxidative damage (29). Superoxide dismutase, oxidoreductases, and heme oxygenase are among the genes regulated by Nrf2 (30). Nrf2 has been shown to have neuroprotective properties in recent investigations (31). Importantly, HO-1 potentially regulates Nrf2 production whereby Nrf2 activation is followed by the increased transcriptional activation of the HO-1 expression (32).

In our research findings, we explored the correlation between recurrent IMI exposures and hippocampus toxicity in mice, as well as the relevance of the Nrf2/HO-1 axis in IMI-induced hippocampus damage. Very few experiments addressed the toxicological effects in the hippocampus caused by IMI exposure in mice; with this background, the present study was undertaken to investigate the histopathological changes, cell death, inflammation, oxidative stress, and the indispensable signaling pathway (Nrf2/HO-1) in the oxidative stress response in various regions of the brain, mostly the hippocampus, following IMI exposure. In addition, we investigated the potential protective effect of AA on imidacloprid-induced neurotoxicity.

## Materials and methods

### Materials

Ascorbic acid was purchased from Sisco Research Laboratories Pvt. Ltd., Mumbai, India and imidacloprid from Insecticide limited, Jammu and Kashmir, India. Kits for assessments of antioxidant biomarkers superoxide dismutase (SOD; CAT No. 19160-1KT-F) and malondialdehyde (MDA; CAT No. MAK085) were purchased from Sigma–Aldrich, India and glutathione peroxidase (GPx; CAT No. G6137-100UN) and ROS (CAT No. MAK142-1KT) were purchased from Sigma–Aldrich Pvt Ltd., Munich, Germany. Mice ELISA kits for the estimation of cytokines tumor necrosis factor-α (TNF-α; CAT No. RAB0477-1KT), IL-6 (CAT No. RAB0309-1KT), IL-2, and IL-4 (Thermo Fischer Scientific; CAT No. 88**-**7711-44) were purchased. The antibodies used in the Western blot study were anti-Nrf2 (CAT No. ab92946), anti-HO-1 antibody (CAT No. ab305290), secondary antibody Goat Anti-Rabbit IgG H&L (HRP; CAT No. ab6721), and Goat Anti-Rat IgG H&L (HRP) preadsorbed (CAT No. ab7097) were purchased from Abcam, India. All other compounds were mostly of importance in research and bought from reputable suppliers.

### Animals

The Institutional Animal Ethics Committee (IAEC) of the National Institute of Pharmaceutical Education and Research (NIPER), India approved the research protocol, and all experiments followed the guidelines set forth by the committee for the purpose of control and supervision of experiments on animals (CPCSEA) in New Delhi, India; IAEC certificate No. NIPER-H/IAEC/10/21. The study used male albino mice of Swiss strain weighing 20–30 g procured from the NIPER, Hajipur, Bihar. Mice were housed in a 25°C air-conditioned area with a 12-h light/dark cycle, kept in appropriate hygiene practices, and supplemented with feed and water.

### Experimental design

The mice were randomly divided into four groups, with six mice in each group. For a period of 28 days, in the first group, mice were given a normal diet, water, and corn oil. The mice in the second group were administered with IMI (45 mg/kg/po/body weight daily for 28 days) dissolved in corn oil in the same dose as previously employed (33). Mice in the third group were treated with IMI plus AA (45 mg/kg/po/body weight plus 200 mg/kg /po/animal body weight per day up to 28 days) dissolved in corn oil and water, respectively, and the mice in the fourth group were treated with AA of 200 mg/kg/po. AA and IMI were dissolved in water and corn oil and given by oral route of administration.

Behavioral tests, such as the Y-maze and new object identification tests were conducted on day 29 of the trial, after which the animals were decapitated. The hippocampus was dissected right away, with one part being prepared for tissue homogenate and another being put in 10% formalin for histological investigation.

All the experiments were carried out according to the procedures of the IAEC of the NIPER in Hajipur, India.

### Behavioral tests

#### Y-maze test

A Y-maze assessment was conducted to assess the spatial recognition memory of mice following IMI and ascorbic acid treatments. The Y-maze device shown in this investigation was made of hardwood, which had tripled uniformly placed arms (20.32-cm long, 7.62-cm height, and 12.7-cm wide) with an equilateral triangular center region. Two trials, namely, the acquisition trial and the retrieval trial, were carried out on the animal. On day 27 of the study, all animals were given their initial learning test (acquisition trial), where they were put inside the center of the Y-maze apparatus and allowed to simply wander around the three arms of the labyrinth for 5 min without reinforcement. To lessen the odor impact, the maze was washed with 70% ethanol and allowed to dry between animals, and the mice have been transferred to respective cages until the second trial (retrieval trial) that was carried out on day 28 of the research (24 h after the last dose of IMI). Entry into an arm was recorded only if the animal's hind paws were totally inserted into the arm (34, 35). A video camera was used to record all the activities to explain the results, which were then manually examined. Spontaneous alternation behavior (SAB) was defined as the entry of the animal into all three arms of the Y-maze on successive decisions. On day 28 of the experiment, the proportion of spontaneous alternation behavior was calculated as follows:


$$\begin{array}{l} \%\;Alteration\;is\;calculated\;as\;number\;of\;alteration/Number\\ of\;entries\;\times \;100 \end{array}$$

#### Novel objects recognition test

This method is designed to assess the animals' cognitive memory and learning. It is based on testing animals' abilities to explore the novel object over the familiar one when both objects are provided at the same time. A square wooden field (40 × 40 cm) serves as the basis for the equipment. The animals were subjected to two sessions (a training session and a retention session). On day 27 of the experiment, for a training session, all mice were transferred into the open field apparatus in the center using two consecutive things (rectangular yellowish wooden bars 4-cm long × 2-cm width), the objects were located in two adjacent corners, 10 cm away from one another and the walls for 5 min, the animals were allowed to move freely to examine both objects. The animals were observed only when the head of the animal was oriented to the object with its nose touched or sniffed the object. After each animal was tested, the objects and apparatus were washed with 70% ethanol to ensure that all olfactory stimuli were removed. The animals had a retention session on day 28 of the experiment (24 h following the training session) in which the familiar object was changed with a new one (the yellow object being exchanged by a triangle blue wooden bar with sizes of 5 × 3 × 3 cm). Overall positioning of the new object was equally balanced among the subjects. All mice were allowed 5 min to investigate, with the recorded time with further assessments.


$$\begin{array}{l} Discrimination\;index\;\left(DI\right)\;=\;The\;time\;spent\;with\;exploring\\ the\;novel\;object/The\;total\;exploration\;time \end{array}$$

### Histopathological examination

The histological assessments were conducted as mentioned in a previous study (12). The brain hippocampus of all mice was decapitated and dissected. Then, they were dehydrated with alcohols after being incubated in 10% formal saline for 24 h. Hippocampus tissues were fixed in paraffin and cut into 5-μm thickness of slices for hematoxylin and eosin staining (H and E). A blindfolded pathologist examined and photographed specific hippocampal regions, namely, CA1 and CA3, and the dentate gyrus (DG), using a light microscope (MDT International, Ambala Cantt, Haryana, India) at a resolution of 400 × (at least three independent samples per group were examined and photographed). Every specimen was given a number indicating the degree of tissue destruction and hippocampal neurotoxicity.

### Hippocampus homogenates preparation

The mice hippocampus was rapidly extracted, weighed on ice, and cleaned in cold saline. Briefly, ~10 strokes in a glass homogenizer (1,200 rpm) homogenized 20% w/v hippocampus tissue in 0.1-M phosphate buffer (PBS), with a pH of 7.4 by cooling centrifuge, and the homogenates were spun at 3,000 rpm for 20 min at 4° temperature. The supernatants were collected and kept at −80°C until they were analyzed.

### Assessment of the oxidative stress techniques

#### Assessment of malondialdehyde

Malondialdehyde was evaluated in hippocampus homogenates using a malondialdehyde kit by Sigma–Aldrich. This method was based on the amount of malondialdehyde, which was measured by 532-nm excitation wavelength and 553-nm emission detection. Microplate readers were used to determine the fluorescence of the colored product. The MDA concentration was measured using the following formula:


$$\begin{array}{l} MDA\left(nmol/ml\right)=\left(S_{\text{a}}/S_{\text{b}}\right)\times D=C, \end{array}$$

where S_a_ = MDA concentration in the unknown sample (nanomole) based on an established curve; S_b_ = the quantity (mg) or sample size (ml) put into the wells; C = MDA concentration in the sample; and D = factor of sample dilution.

#### Assessment of ROS

According to a previous study (36), ROS generation was measured using the 2′,7′-dichlorodihydrofluorescein diacetate (H_2_DCFDA) dye method based on the ROS-dependent oxidation of Dichloro-dihydro-fluorescein diacetate (DCFH-DA) to Discounted Cash Flow (DCF). An aliquot of 100-μl hippocampus homogenate was transferred to a 96-well plate and incubated with 10 μl of 10 mmol H_2_DCFDA for 15 min in the dark. The H_2_O_2_, OH^−^, and ONOO^−^ produced during the cellular oxidative response oxidized the non-fluorescent intracellular DCFH into the highly fluorescent DCF. Furthermore, DCF fluorescence was assayed at 530 nm after the excitation of cells at 488 nm. Acquisition and analysis of the processed sample were performed on a Synergy H1 microplate reader (GEM 5 software, BioTek Company). H_2_DCFDA dye was used to measure ROS. To begin, 100 μl of hippocampus homogenate was extracted from each sample in a 96-well plate, 2 μl (1 mM) of dye was applied to each sample, and the 96-well plates were kept at 37°C for 30 min before reading the fluorescent. Then, using a microplate reader, we observed fluorescence at excitation of 504 nm and emission of 530 nm, and the number of folds that changed across groups was also determined (37).

#### Assessment of superoxide dismutase

The SOD expression in hippocampus homogenates was assessed by using a new method (superoxide dismutase ELISA kit by Sigma–Aldrich, India). The assay is based on SOD's ability to block the reduction of nitro blue tetrazolium dye by phenazine methosulphate. A sample with a volume of 0.02 ml was mixed with a 0.2 ml (50 mM/L phosphate buffer, pH 8.5, 1 mM/L) experimental solutions and nitro blue tetrazolium (1 mM/L NADH) solution in a 1:10:1 mL ratio. To start the reaction, 0.02-mL enzyme working solution was added to the mixture. After incubating the plates at 37°C ~20 min, an absorption spectrum of 450 nm was recorded. The following formula was used to calculate SOD activity (percentage inhibition rate):


$$\begin{array}{l} SOD\;activity\left(U/g\;tissue\right)=\left(A_{\text{blank}}1-A_{\text{blank}}3\right)\\ -\left(A_{\text{sample}}-A_{\text{blank}}2\right)/\left(A_{\text{blank}}1-A_{\text{blank}}3\right)\times 100 \end{array}$$

#### Assessment of glutathione peroxidase

Using a glutathione peroxidase assay kit from Bio-Diagnostic, the activity of glutathione peroxidase in hippocampus homogenates (kit by Thermo Fischer Scientific, India) was assessed. The technique is based on an indirect assessment of glutathione peroxidase activity, in which glutathione peroxidase reduces an organic peroxide to create oxidized glutathione (GSSG). Glutathione reductase uses NADPH as a cofactor to recycle this product to its reduced state. The protocol was followed according to the instructions provided by the manufacturer. The enzyme activity (μmole/mg of protein) is calculated using the following formula:


$$\begin{array}{l} GSSG=Total\;GSH-Free\;GSH/2 \end{array}$$

#### Assessments of IL-2 and IL-4

The activities of interleukin-2 (IL-2) and IL-4 in hippocampus homogenates were measured using IL-2 and IL-4 ELISA kit by Thermo Fischer Scientific, India. Antigen and antibody reactions are used in the experiment. The wavelength of the colored product was determined using spectrophotometry at 550 nm when the reaction was completed (38).

#### Assessment of IL-6

The activities of IL-6 and TNF-α in the sample of hippocampus homogenates were determined using the ELISA kit from Sigma–Aldrich, India (IL-6 and TNF-α ELISA kit). This ELISA was designed to measure IL-6 using the biotin double antibody sandwich technique. To assess IL-6, the IL-6 monoclonal antibodies were incubated, followed by the addition of anti-IL-6 antibodies labeled with biotin to combine with streptavidin-HRP, which forms an immune complex. After incubation, the unbound enzyme was washed. When substrate A was added to substrate B, the acid's impact caused the solution's color to change from blue to yellow. Positive correlations were found between the mouse IL-6 concentration and the solution's color intensity (38).

#### Assessment of TNF-α

TNF-α activity in hippocampus homogenates was measured using an ELISA (TNF-α ELISA kit from Sigma–Aldrich, India) in accordance with the manufacturer's recommendations. This ELISA was designed to measure TNF-α using the biotin double antibody sandwich technique. To assess TNF-α, the TNF-α monoclonal antibodies were incubated, followed by the addition of anti-TNF-α antibodies labeled with biotin to combine with streptavidin-HRP, which formed an immune complex. After incubation, the unbound enzyme was washed. When substrate A was added to substrate B, the acid's impact caused the solution's color to change from blue to yellow. Positive correlations were found between the mouse TNF-α concentration and the solution's color intensity (38).

#### Assessment of acetylcholinesterase activity in the hippocampus and the frontal cortex

The activity of acetylcholinesterase was evaluated using acetylcholine iodide as a substrate and 5,5'-dithiobis-2 nitro benzoic acid (DTNB) as the coloring material (39). Phosphate buffer was present in 1.0 ml of the reaction mixture (0.1 M, pH 7.4). DTNB and reacted combination was added 0.1 mL of acetylthiocholine iodide. Subsequently, the wavelength of the absorbance was measured using a spectrophotometer (412 nm) (10 min), the rate of breakdown of acetylcholine iodide, were determined, and the results were represented in μmoles/min/mg protein.

### Measurements of HO-1 and Nrf2 by Western blot

All cryogenic tissue samples were homogenized and processed in an ice-cold lysis solution containing a protease inhibitor cocktail (10% glycerol, 2% SDS in 62 mM Tris-HCl, pH 6.8; Sigma–Aldrich Ltd.). The protein concentration of the protein lysates was determined using the Bradford technique. Under denaturing conditions, equal quantities of protein content were resolved by sodium dodecyl sulfate polyacrylamide gel electrophoresis (SDS-PAGE) and transferred to nitrocellulose membranes. After blocking with 6% non-fat dry milk in TBS-Tween buffer for 3 h at 4°C, the nitrocellulose membranes were incubated overnight at 4°C with the primary antibodies against the target proteins (Nrf2, HO-1). We utilized human/mouse/rat Nrf2 antibody monoclonal mouse from Abcam for Nrf2 and mouse/rat antibody from Abcam for HO-1 (Abcam).

### Statistical analysis

The data were analyzed by GraphPad Prism version 9. The differences between groups were calculated using a one-way analysis of variance (ANOVA) followed by the Tukey–Kramer multiple comparisons test. Data were expressed as mean ± SEM; a *p*-value of < 0.05 was considered statistically significant.

## Results

### AA improves neuronal impairments in IMI exposure animals

**Figure 1:**
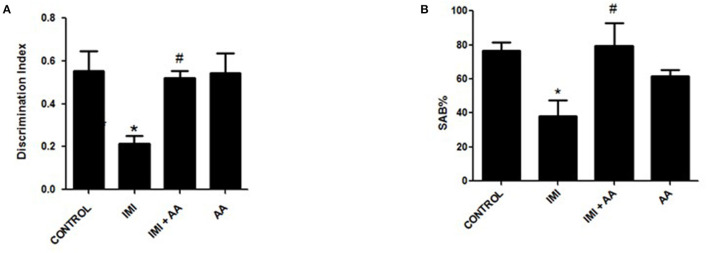
Y-maze and novel target recognition behavioral tests. Control, IMI, IMI+ Ascorbic acid, and Ascorbic acid treated animals were subjected to Y- maze **(A)** and Novel target recognition test; **(B)** Discrimination index; after 28 days of IMI daily doses. SAB, Spontaneous Alteration Behavior; DI, Discrimination Index. Data represent mean ± SEM. *n* = 6/group. *Represents significant values when compared to controls at *p* < 0.05, ^#^represents significant values when compared to IMI-treated animals at *p* < 0.05.

We conducted two behavioral assessments on the mice to evaluate their neuronal impairments. To evaluate the mice's short-term working memory, a Y-maze experiment was performed after repeated IMI treatment. In this test, the spontaneous alternation behavior (SAB) was determined, as explained in the “Materials and Methods” section. The Y-maze test results showed that IMI-treated mice exhibited significantly reduced SAB compared to normal control mice. In contradiction to IMI-treated mice, AA treatment dramatically enhanced SAB in IMI + AA-treated animals (Figure 1A).

This innovative target tracking technique was used to assess the cognition memories of mice. According to recent findings, IMI treatment impaired the animals' cognition and reduced the amount of time they spent exploring a novel object compared to exploring a recognized object. According to the retention phase results, the IMI-treated animals' discriminate score (DS) was significantly lower than the score of the control mice. In comparison to the familiar object, the group of IMI + AA-treated mice spent considerably more time studying the unfamiliar object. Despite their exposure to IMI, the mice treated with AA showed an increase in curiosity and recognition. Overall, the results of the behavioral tests showed that, after IMI exposure, AA improved both spatial and non-spatial memory abilities in mice (Figure 1B). In general, the findings of behavioral tests indicated AA improved animal spatial and non-spatial memory functions in mice followed by an IMI treatment.

### AA protecting the hippocampal of the hippocampus against IMI-induced damages

**Figure 2:**
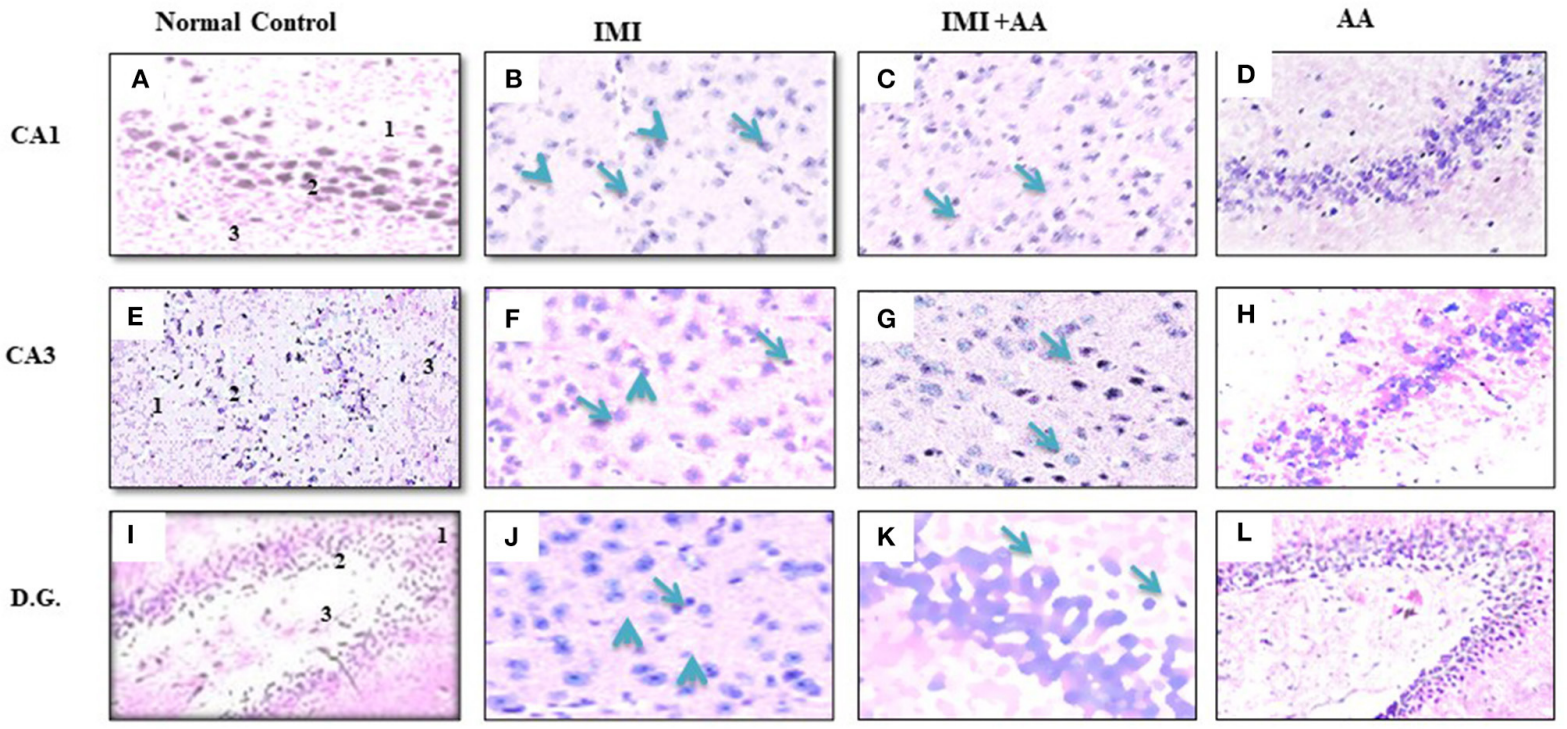
Histopathological assessments of the brain hippocampus areas. **(A, E)** CA1 and CA3 hippocampal regions control mice indicates normal morphology of hippocampus layers (superficial polymorphic layer “1,” middle pyramidal cells layer “2,” and inner molecular layer “3”) with regularly arranged intact neurons. **(B, F)** CA1 and CA3 hippocampal areas of IMI treated mice indicates severe disarrangement of pyramidal cell layers, many apoptotic cells with dark blue stained nuclei and apparent a high number of glial cells (arrowheads). **(C, G)** CA1 and CA3 hippocampus regions of IMI+ Ascorbic acid treated mice respectively show more organized pyramidal cells, apparent intact neurons (arrow) scattered among damaged shrunken neurons with pyknotic nuclei (arrow), and mild glial cells infiltration. **(D, H)** CA1 and (CA3) regions of Ascorbic acid treated mice represents normal and healthy cells at hippocampal layer with regular intact neurons. **(I)** Dentate Gyms hippocampal region of control mice demonstrated normal morphological architecture (molecular layer “1,” granular layer “2,” and hilum “3”) with well-organized neurons bearing intact nuclei. **(J)** Dentate Gyrus of IMI treated mice shows disorganized granular layer with severe edema (arrow), many apoptotic cells (dotted arrow), shrinking hilar cells (arrowhead), and increased number of glial cells. **(K)** Dentate Gyms of IMI+ Ascorbic acid treated mice shows more organized hippocampal zones with moderate edema (arrow), many apoptotic cells (dotted arrows), and many glial cells are still evident. **(L)** Dentate Gyms of Ascorbic acid treated mice shows organized hippocampal zones (400 × magnification).

H and E staining had been performed to evaluate the architectures of the hippocampal areas, primarily CA1 and CA3, as well as the dentate gyrus (DG), to observe if AA might prevent the hippocampus from the damage inflicted by neonicotinoids IMI. In comparison to controls, imidacloprid treatment caused moderately severe impairment toward the CA1, CA3, and DG hippocampal areas in terms of neurodegeneration, gliosis, and edema. Furthermore, IMI-treated mice hippocampal neuron exhibited the following: a significant disorder of neuronal cell layers with degeneration and shrinkage in the CA1 area; the emergence of numerous apoptotic cells alongside elevated microglia, mostly in CA3 zone; and severe edema with a reduction of hilar cells inside the DG area (Figures 2B, E, H). Significantly, IMI + AA-treated animals had mild-to-moderate hippocampus damage compared to IMI-treated mice (Table 1). Both CA1 and CA3 areas of the IMI + AA-treated mice had better-structured neurons, having visible surviving neurons dispersed among many apoptotic and microglial cells. Moreover, as compared to IMI-treated mice, the DG area of IMI + AA-treated mice revealed more ordered zones with considerable edema (Figures 2C, F, I). Our findings suggest that AA may be capable of protecting mouse hippocampus areas against imidacloprid-induced damage.

**Table 1:** Histopathological score of hippocampus regions (CA1, CA3, and dentate gyrus) in control, IMI, IMI +AA, and AA.

**Group**	**Control**			**IMI**			**IMI** + **AA**			**AA**		
**Region parameter**	**CA1**	**CA3**	**DG**	**CA1**	**CA3**	**DG**	**CA1**	**CA3**	**DG**	**CA1**	**CA3**	**DG**
Neuronal degeneration	**-**	-	-	**++**	**++**	**+++**	**+**	**++**	**++**	**++**	**+**	**+++**
Edema	-	-	-	**++**	**++**	**+++**	**++**	**++**	**++**	**+**	**+++**	**++**
Gliosis	-	-	**+**	**+++**	**++**	**++**	**++**	**++**	**++**	**+**	**++**	**++**

-, Absent lesions; +, mild lesions; ++, moderate lesions; +++, severe lesions. N = 3 for each region per group.

### AA protects the upregulation of acetylcholinesterase activity

**Figure 3:**
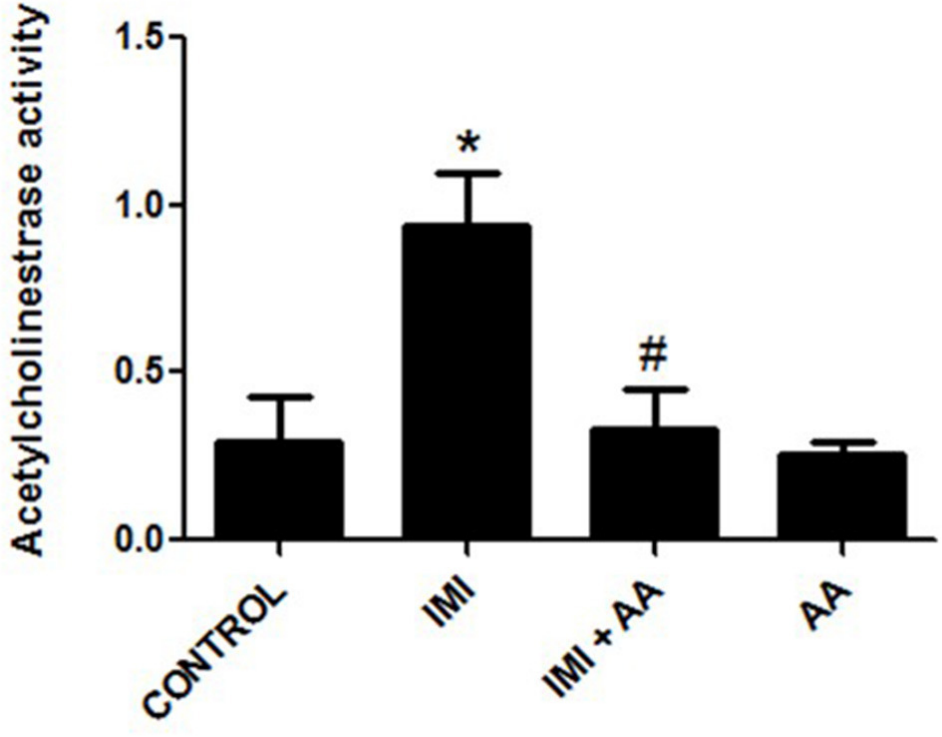
The acetylcholinesterase activity in all studied groups. AchE activity was measured in controls, IMI, IMI+ Ascorbic acid, Ascorbic acid treated animals. Data represent mean ± SEM. *n* = 6 for each group. ^*^Represents significant values when compared to controls at *p* < 0.05, ^#^represents significant values when compared to IMI-treated animals at *p* < 0.05 (IMI, Imidacloprid).

IMI is believed to cause toxicity by decreasing the action of acetylcholinesterase (AChE). As a result, we used Ellman's approach to assess the AChE activity in hippocampus tissue homogenates. The present study found that repeatedly exposing mice to a subthreshold dosage of IMI (45 mg/kg/po/body weight) for 28 days did not trigger acetylcholine acute toxicity such as seizures, diarrhea, and muscle stiffness as explained in a previous study. Acetylcholinesterase activity was higher in IMI-treated mice than in controls. However, AA therapy prior to IMI exposure significantly reduced the inhibition of AChE activity relative to IMI-treated mice. The ability of AA to maintain the AChE activity suggests that it may have therapeutic promise in reducing AA-induced neurotoxicity (Figure 3).

### AA protective role against lipid peroxidation and oxidative stress caused by IMI

**Figure 4:**
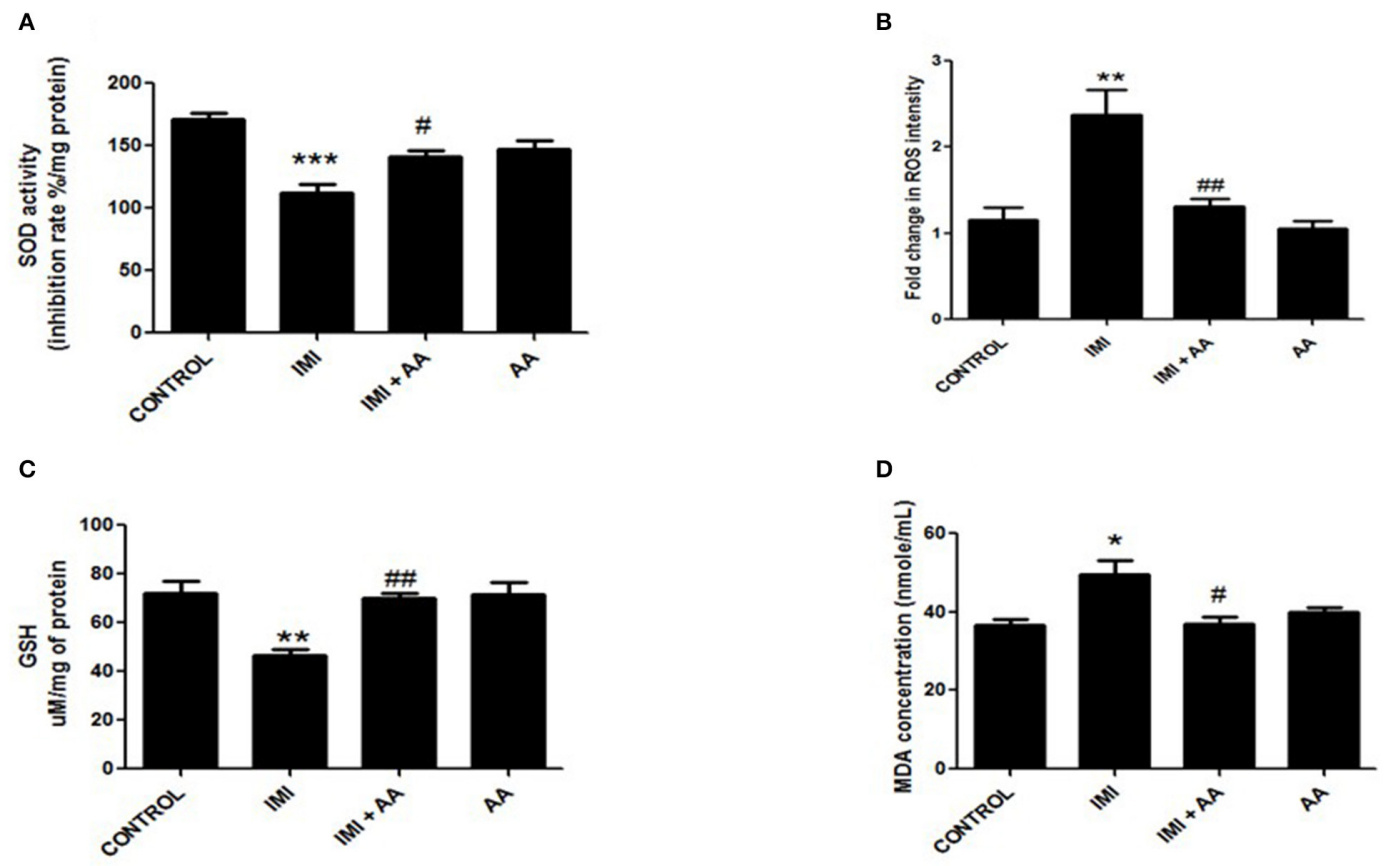
The effect of Ascorbic acid cm oxidative stress biomarkers. Column figure shows the oxidative stress biomarkers measured in whole brain homogenates of control, IMI, IMI+ Ascorbic acid, and Ascorbic acid treated animals. **(A)** Superoxide Desmutase; **(B)** Reactive oxygen species intensity; **(C)** Glutathione activity; **(D)** *Malondialdehyde* (MDA) represent mean ± SEM. *n* = 6 for each group. ^−^Represents significant values when compared to controls at *P* < 0.05, ^#^represents significant values when compared to IMI-treated animals at *P* < 0.05, ^##^represents significant values when compared to IMI-treated animals at *P* < 0.05. ^*^P ≤ 0.05, ^**^P ≤ 0.01, ^***^P ≤ 0.001. These data represent the significant different between treatment and disease control group.

IMI has an oxidative stress pathway stimulation, which has negative impacts on cell components. In stressful situations, reactive oxygen species (ROS) encourage the formation of large levels of malondialdehyde (MDA). As a defense mechanism against ROS, the cell boosts the synthesis of antioxidant enzymes such as glutathione peroxidase and superoxide dismutase. Furthermore, the data demonstrated that IMI-treated mice had greater MDA and ROS levels than control mice. Furthermore, IMI-treated mice had considerably reduced antioxidant enzyme activity than control mice. Surprisingly, the hippocampus contents of MDA and ROS in IMI + AA-treated mice were considerably lower than those in IMI-treated mice. Furthermore, IMI + AA-treated mice had considerably greater activities of antioxidant enzymes than IMI-treated mice (Figure 4). These findings suggest that, when supplied throughout IMI treatment, AA efficiently maintains the activity of antioxidant enzymes and protects against lipid peroxidation.

### AA protects against the IMI-induced neuroinflammation

**Figure 5:**
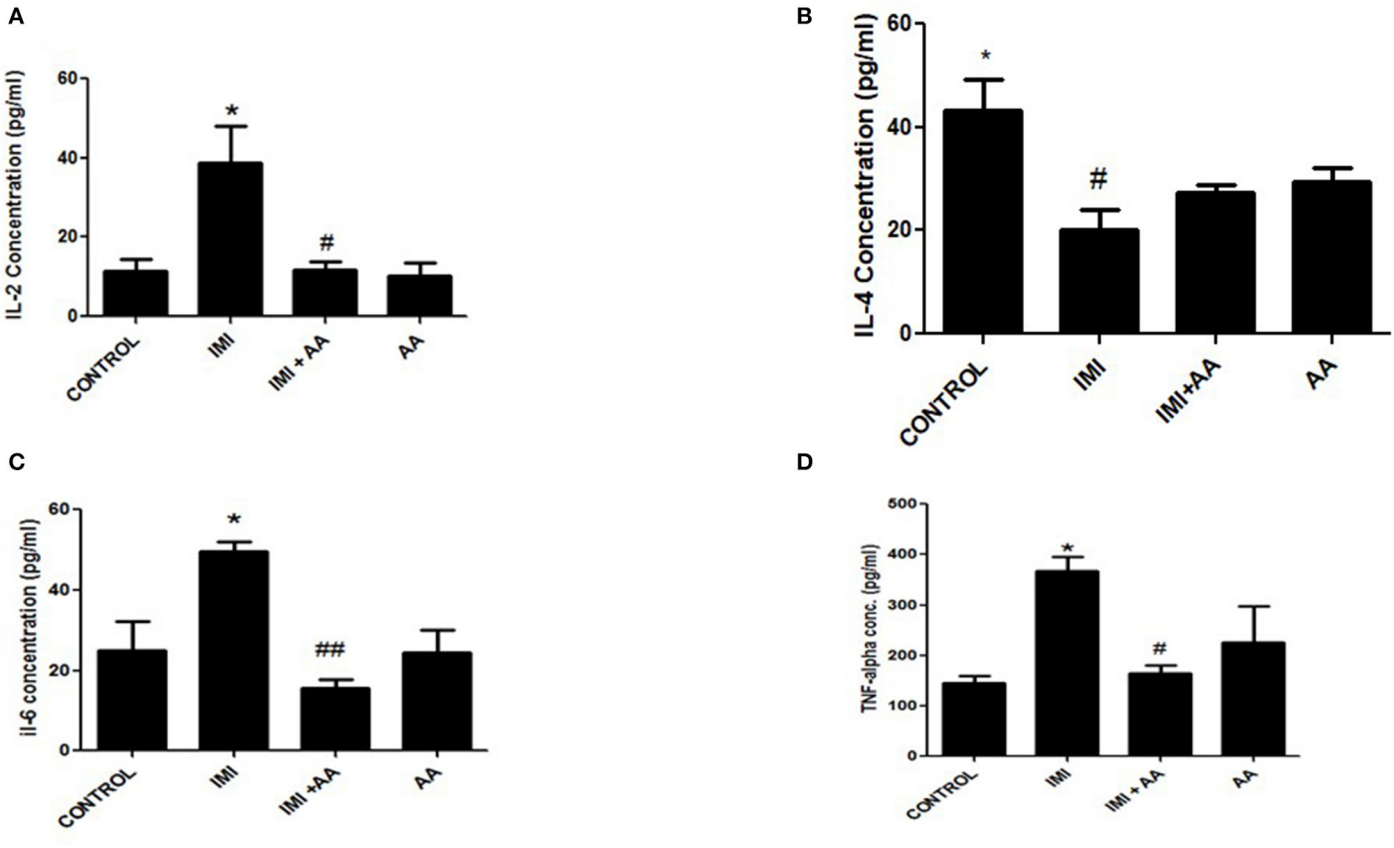
The effect of Ascorbic acid on inflammatory biomarkers. Column figure shows the inflammatory biomarkers measured in whole brain homogenates of control, IMI, IMI+ Ascorbic acid, and Ascorbic acid treated animals. **(A)** Interleukins-2; **(B)** Interleukins-4; **(C)** Interleukins-6; **(D)** TNF- alfa. Data represent mean ± SEM. *n* = 6 for each group. ^*^Represents significant values when compared to controls at *P* < 0.05, ^#^Represents significant values when compared to IMI-treated animals at *P* < 0.05. ^##^*P* ≤ 0.01. These data represent the significant different between treatment and disease control group.

Inflammatory mediating proteins overexpression (TNF-α, IL-2, and IL-6) were significantly raised by IMI exposure (Figures 5A, C, D). IL-4 essential anti-inflammatory mediators, whose expression increased inside the low and intermediate IMI dosage groups, were inhibited in the higher IMI dose range (Figure 5B), which had been enhanced by administering AA compared to the high IMI dose group.

### AA exerts its neuroprotective effects *via* Nrf2/HO-1 pathway

**Figure 6:**
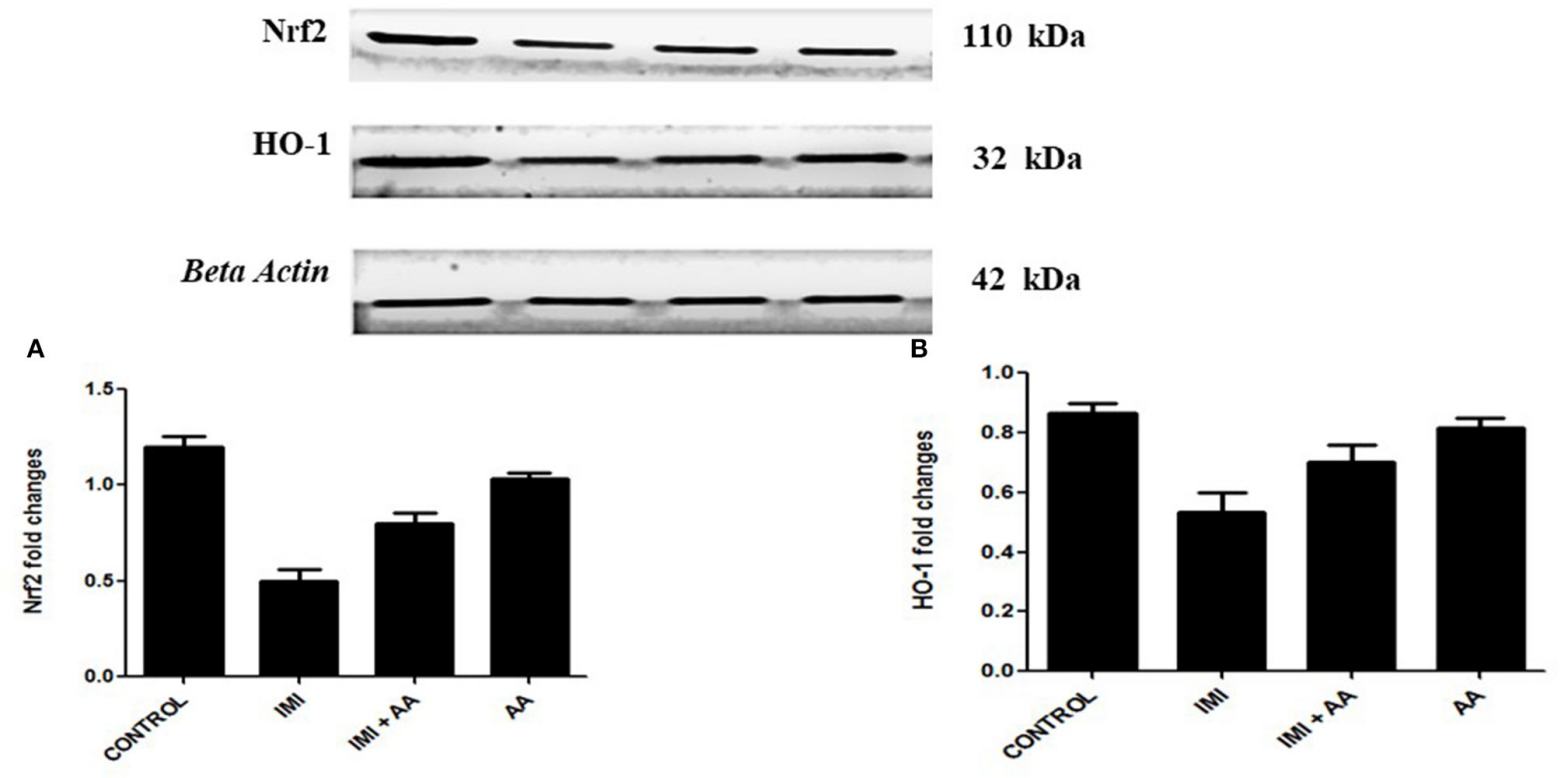
Western blot analysis for Nrf2, heme oxygenase-1 **(A)** Western blots for the measured factors in comparison to β-actin, lane 1 (control), lane 2 (IMI treated), lanes 3 (Ascorbic acid + IMI (200 mg/kg), and lane 4 (Ascorbic acid (200 mg/kg). Column charts for fold changes in Nrf2. **(A)**, heme oxygenase-1 **(B)**. IMI, Imidacloprid; AA, Ascorbic acid; HO-1, heme oxygenase-1 Data are the mean ± SD, and analysis was done by one-way ANOVA followed by Tukey's *post-hoc* test. (a) *P*-value < 0.05 compared to the control group, (b) *P*-value < 0.05 compared to the IMI group, and (c) *P*-value < 0.05 compared to the AA (200 mg/kg) group.

The Western blot was used to perform the expression levels of H0-1 and Nrf2 genes to better understand the molecular mechanism by which AA can protect hippocampus cells. Because prolonged oxidative stress causes a decrease in nuclear HO-1, the HO-1/Nrf2 pathway is a critical target for oxidative stress. Indeed, our results showed that IMI-treated mice exhibited significantly lower expression levels of HO-2 and Nrf2 compared to the controls. These results refer to the involvement of HO-1 pathway in the oxidative stress induced by IMI exposure (Figure 6). Moreover, AA might affect this particular pathway to exert its neuroprotective effects.

## Discussion

Throughout the ongoing investigation, we observed that recurrent IMI administration was highly associated with neurotoxicity, oxidative stress, and memory deficits in mice. Furthermore, taking AA with IMI may protect against these consequences, potentially *via* modulating the Nrf2/HO-1 pathway. Our research findings are consistent with prior research, which found that exposure to organophosphorus insecticides is highly associated with neurodegenerative and behavioral changes (40). Remarkably, the cytotoxicity of these chemicals is recognized not only in acute high levels of exposure but also in recurrent low levels of exposure (41). Researchers discovered that repeated exposure to IMI dosages manifested in deficiencies in attentiveness, neurotoxicity, and impulsive behavior in mice. Furthermore, despite the recovery of acetylcholinesterase activity after a washout period, these behavioral alterations persisted. In this investigation, we were using a moderate IMI dosage (45 mg/kg/po for 28 days) to investigate the histopathological and signaling pathways alteration induced by repeated low IMI dose.

IMI neurotoxicity is mostly induced by oxidative stress and acetylcholinesterase activation. Because cholinergic neurotransmitter is essential in neurodevelopment, interfering with AChE activity disrupts the amplitude of neurodevelopmental mechanisms, thereby affecting neuronal survival, multiplication, synaptic function, and behavior (42). Chronic exposure to IMI, according to research observations, dramatically increases AChE activity and is linked with significant impairment, including both spatial and non-spatial memory performance. While AChE activation is believed to be just the primary mechanism generating IMI neurotoxic, other non-cholinergic pathways play a role in both acute and long-term toxicity (43). For example, an alteration of cellular components such as tubulin, impairment of mitochondrial dynamics, and glutamate-mediated excitotoxicity has been proposed (44, 45). While findings from some studies revealed Ca homeostasis and elevated production of inflammatory mediators, several investigations have shown that IMI can significantly modify the serine hydrolase, neurotrophin, cannabinoid, and muscarinic receptor activities (46, 47). IMI was previously demonstrated to cause oxidative stress in several organs, including mouse and rat kidney, liver, and central nervous system (48, 49). Several studies have already shown that antioxidant compounds can help reduce the production of free radicals as well as the oxidant involved in IMI cytotoxicity. Interestingly, IMI cytotoxicity may be reduced by utilizing protective vitamins such as vitamin E (50). In addition to a therapy utilizing natural antioxidants such as turmeric and thymoquinone has been employed as a potential therapeutic entity against various neurodegenerative and neurotoxicity conditions (51).

We investigated the HO-1/Nrf2 signaling pathways associated with oxidative stress to determine the molecular basis through which AA exhibits its neuroprotective effects. We also measured the level of glutathione peroxidase or reactive oxygen species to correlate the relation with oxidative stress, inflammation, and cell death, because these pathways are highly associated with oxidative stress circuits, which may have been affected after IMI neurocytotoxicity. Oxidants cause HO-1 stimulation as well as a decrease in nuclear Nrf2 (52, 53). Notably, oxidative stress has a role in the progression of memory deficit caused by various oxidants (54). Upregulation of HO-1, in particular, is implicated by elevated phosphorylation and accumulation and, hence, is associated with Alzheimer's disease pathogenesis. Furthermore, because HO-1 stimulation through the Nrf-2 route in the hippocampus performs important roles in hippocampus injury prevention, there, really, are various HO-1 inducers/modulators for medication or prospective therapeutic functions, such as traditional remedies (55). Our findings pertain to the involvement of oxidative stress, namely the HO-1/Nrf2 pathways, on learning and memory. Our findings are consistent with a prior study that discovered that prolonged rat exposure to antioxidants such as edaravone resulted in depressive and neurological disorders *via* over expression of HO-1/Nrf2 (56). Moreover, our results are in line with the potential research findings, in which they discovered a link among ROS generation and Nrf2 activity and hypothesized the Nrf2 activity, which declines with the course of neurodegenerative disorders. Surprisingly, combining AA with IMI might effectively mitigate the severity of these transcriptional alterations. Our findings clearly implied that the HO-1/Nrf2 pathway is one of the mechanisms that AA targets in its antioxidant as well as neuroprotective activities. Especially, our findings are similar to those of a previous study, which revealed how vanillic acid antioxidants might reduce synaptic disorganization and neuronal impairment in A1-42 mice treated, as shown by enhanced performance in Morri's water maze and Y-maze tests (57). Such improvements were achieved by increasing the HO-1 activity *via* a mechanism that involves the HO-1/Nrf2 pathways (58).

AA is an essential antioxidant that has previously been shown to fight against hyperglycemia, cardiovascular problems, lung non-small cell carcinoma, and colorectal cancer (59–61). For the first time, the current investigation found that AA had a substantial protective impact against IMI-induced neurotoxicity. Our findings demonstrated that AA was beneficial in reducing AChE activity and oxidative stress, cell death *via* oxidative stress, and modulated the Nrf-2 and HO-1 signaling cascades as well as in preventing neuronal toxicities. Such pharmacological findings could be utilized as a neurotoxicity-protective therapeutic entity.

According to our research and literature findings, we can conclude that repeated exposure of imidacloprid causes oxidative stress and memory impairments in mice. While the current study indicated that AA ameliorates IMI-induced various neurological and neurotoxicity in mice, it has therapeutic potential to reduce IMI-induced neuronal toxicity and hippocampus toxicity, *via* the possible mechanism of the HO-1/Nrf2 signaling pathway.

## Data availability statement

The original contributions presented in the study are included in the article/supplementary material, further inquiries can be directed to the corresponding author.

## Ethics statement

The Institutional Animal Ethics Committee (IAEC) of the National Institute of Pharmaceutical Education and Research (NIPER) in Hajipur, India approved the research protocol (IAEC certificate No. NIPER-H/IAEC/10/21), and all experiments followed the guidelines set forth by the Committee for the Purpose of Control and Supervision of Experiments on Animals (CPCSEA), New Delhi, India.

## Author contributions

RM had the idea for the article. SSh performed the literature search, data analysis, and drafted the manuscript. RM, SSh, and SSi edited and critically revised the work. All authors contributed to the article and approved the submitted version.
